# Sequence Analysis of Different Domains of *Plasmodium vivax* Apical Membrane Antigen (PvAMA-1 gene) Locus in Iran

**Published:** 2012

**Authors:** A Motevalli Haghi, M Nateghpour, GhH Edrissian, Z Sepehrizadeh, M Mohebali, MR Khoramizade, S Sabouri Shahrbabak, H Moghimi

**Affiliations:** 1 Dept. of Medical Parasitology and Mycology, School of Public Health, Tehran University of Medical Sciences, Tehran, Iran; 2 Center for Research of Endemic Parasites of Iran (CREPI), Tehran University of Medical Sciences, Tehran, Iran; 3 Dept. of Pharmaceutical Biotechnology, Pharmacy faculty, Tehran University of Medical Sciences, Tehran, Iran; 4 Dept. of Pathobiology, School of Public Health, Tehran University of Medical Sciences, Tehran, Iran; 5 Dept. of Microbiology, Faculty of Biology, Tehran University, Tehran, Iran

**Keywords:** *Plasmodium vivax*, Apical membrane antigen, Iran

## Abstract

**Background:**

*Plasmodium vivax* is responsible for approximately 80 million malaria cases in the world. Apical membrane antigen1 (AMA-1) is a type I integral membrane protein present in all *Plasmodium* species. AMA-1 interferes in critical steps of invasion of human hepatocytes by sporozoites and red blood cells by merozoites and is one of the most immunodominant antigens for eliciting a protective immune response in human. It is considered as a promising antigen for inclusion in a vaccine against *P. vivax*. Since more knowledge is needed to lighten the scope of such antigen we compared genetic variation in *P. vivax* AMA-1from an Iranian isolate with those reported from some of the other malarious countries so far.

**Methods:**

*P. vivax* genomic DNA was extracted from the whole blood of an Iranian patient with patent *P. vivax* infection. The nucleotide sequence for 446 amino acid (AA) residues (42–488 of PvAMA-1) was amplified by PCR and cloned in pUC19 vector for sequencing.

**Results:**

Sequence analysis of the antigen showed a high degree of identity (99%) with strong homology to the PvAMA-1 gene of *P. vivax S3* and *SKO814* isolates from India and Korea (Asian isolates) respectively, and 96% similarity with *P. vivax Sal-1* AMA-1 gene from El Salvador.

**Conclusions:**

We cloned and characterized three domains of PvAMA-1 gene from an Iranian patient. Predicted protein sequence of this gene showed some discrepancies in corresponding protein in comparing with similar genes reported from other malarious countries.

## Introduction

One of the most widespread human malaria parasites is *Plasmodium vivax* and about 2.6 billion people are at risk of the infection in the world (1). The infection causes approximately 132-391 million episodes of disease each year (2). Although *P. vivax* is the main responsible for malaria morbidity outside Sub-Saharan Africa, it has received little attention and limited funds for research and control owing to producing less severity than *P. falciparum* (3). Moreover, non-cultivable nature of *P. vivax* has induced some limitations for the study of its molecular biology up to now. Indeed, increasing the knowledge of genetic diversity and population structure of this parasite will result in the development of an effective vaccine to control the disease (4). Apical membrane antigen (AMA-1) is a member of a group of molecules which are quite conserved in *Plasmodium* species (5) and are expressed on the micronemes and surface of merozoite in all *Plasmodium* species (6). AMA-1 is expressed in the late schizont stage of the asexual plasmodia life cycle (7). The antigen plays a unique role in critical stage of invasion the human hepatocytes by sporozoites (8) and red blood cells by merozoites (9–12). This gene shows few predominant haplotype and displays very limited genetic diversity within any geographic region so more investigations are needed to confirm these observations (13).

Moreover, AMA-1 is one of the most immunodominant antigens which are considered as promising antigen for the development of a recombinant vaccine against *P. vivax* (7, 14, 15). It could be also used for immunological studies to evaluate the natural acquired antibody response to *P. vivax* AMA-1 (16). Therefore, polymorphism investigation of this gene in different areas could result in developing diagnostic kits.

Iran is one of the endemic areas for *P. vivax*. The parasite possesses the most reported cases of the malaria parasites each year in the country (17). So it is important to investigate the molecular nature of the parasite nested in Iran in comparing with the same species of the parasites in the other malarious areas. Because of strong candidate of PvAMA-1 for vaccine development and producing diagnostic kits, the PvAMA-1 was sequenced and then compared with similar genes recorded in GenBank.

## Materials and Methods

### Parasite sample

The case was an Iranian inhabitant of Jask district (Hormozgan Province) with symptomatic *P. vivax* infection. Blood of the patient was collected into the EDTA tube using venipuncture method. *P. vivax* infection was documented by microscopic analysis using Giemsa stained thick and thin blood smears.

### DNA extraction

The genomic DNA was extracted from the whole blood using Genomic DNA Extraction Kit, ACCUPreP^®^ kit (BIONEER) according to the manufacturer's instructions. The quality and quantity of the extracted DNA was checked using a biophotometer (Ependorf) at 260 and 280 nm and electrophoresis on 1% agarose gel.

### PCR amplification of PvAMA-1 gene

A partial region of PvAMA-1 gene, including domain I, domain II and domain III, covering amino acids (AA) residue 42–488 was targeted for amplification. The primers were designed based on the sequence of *P. vivax Sal-1* apical merozoite antigen- 1 (PvAMA-1gene, ACCESSION NO.: XM_001615397) with the following sequences: PvAMAF (5′-CCATGGGGCCTACCGTTGAGAGAA-3′) and PvAMAR as (5′- CTCGAGTCATAGTAGCATCTGCTTGTT- 3′).

The PCR was performed in a 200 µl thin-walled PCR tube containing: 200 ng extracted DNA as template, 20 pmol of each primer, 12.5 µl of Pwo master PCR master mix (Roche) and ddH2O up to 25µl. The targeted gene was amplified for 30 cycles (95^°^C for 20s, 58^°^C for 30s and 72^°^for 2 min) and the PCR product was checked on 1.0% agarose gel against DNA size marker (Fermentase), then it was purified from agarose gel by Qiaquick gel extraction kit (Qiagen) to be prepared for cloning.

### Cloning and sequencing of the PvAMA-1 gene

Purified DNA fragment was inserted into a SmaI cut pUC19 plasmid using rapid DNA ligation kit (Roche) according to the manufacturer's protocol. The transformation was done in CaCl_2_ treated competent cells of *Escherichia coli* Novablue using thermal shock method (30min at 0°C, 2min at 42°C and 2min at 0°C). The transformants were incubated at 37°C for 45 min and cultured on the LB agar plates containing 100 µg/ml ampicillin, 1 mM IPTG and 50 µg/ml X-gal and incubated for 16 hours at 37^°^C. White (positive) colonies were selected and validated by PCR. One of the positive clones was cultured in LB medium containing 100µg/ml ampicillin for plasmid purification by using Plasmid Purification Kit (Roche). The extracted plasmid was sent for sequencing using universal M13-pUC primers. DNA sequence was performed by Gen Fanavaran Company (Tehran, Iran). The sequencing result was analyzed by BLAST and ClustalW softwares to compare the reported sequences in GenBank.

## Results

**Fig. 1:**
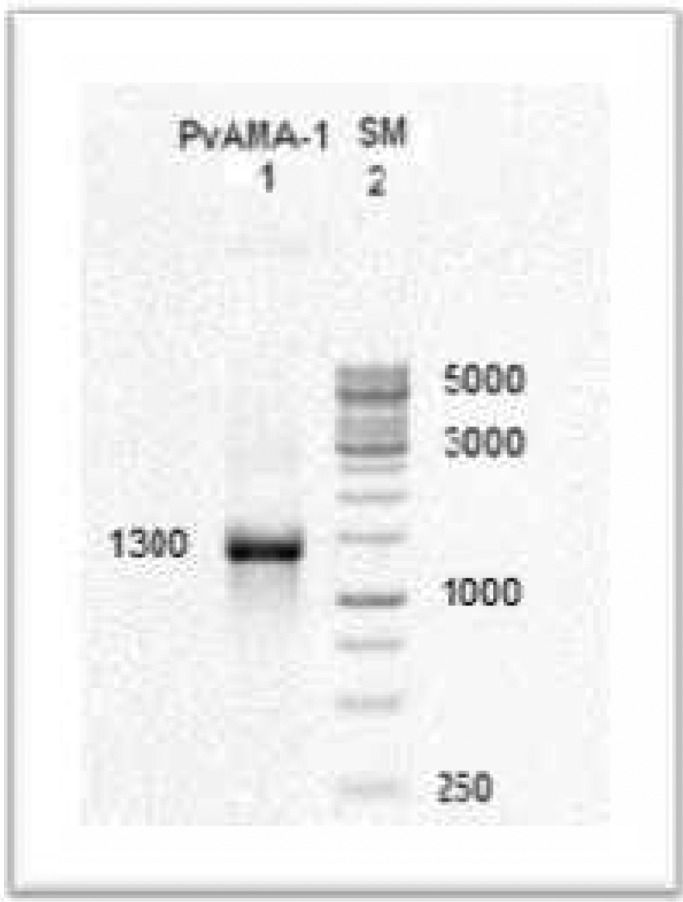
Gel electrophoresis of PCR product from a *P. vivax*-infected patient. Lane1: *PvAMA-1* gene was approximately 1300bp long. Lane2: SM: Size marker

**Fig. 2:**
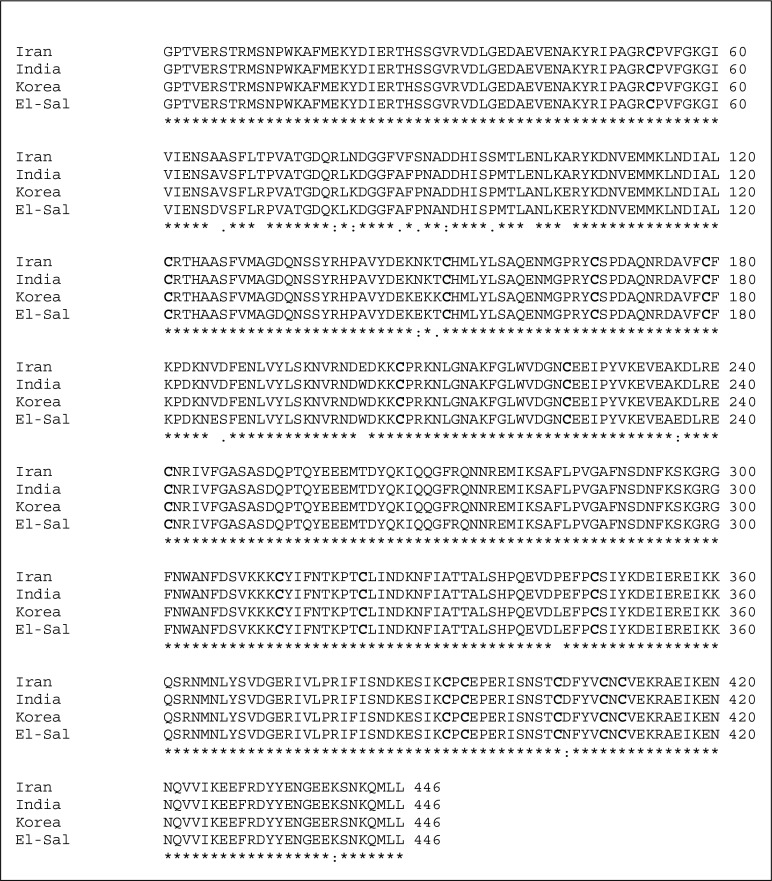
Multiple protein sequences alignment of PvAMA-1 gene in *P. vivax* isolated from Iran and corresponded part of the genes isolated from India, Korea and El Salvador. The great numbers of changes are situated in the most variable domain I of AMA-1. Domain II shows limited diversity in our study. Sixteen Cysteine residues are bold

As Fig. 1 shows a 1300bp band has been resulted from extracted genomic DNA by PCR. The production of PCR was cloned in pUC19 and then sequenced. The sequenced gene was submitted to the GenBank and registered under the HM535663.1 accession number. The predicted protein sequence analysis revealed that it composes of 446 amino acids including 3 domains and sixteen Cysteine residues (Fig. 2).

## Discussion

In the first study on PvAMA-1 that was conducted by Cheng and Saul two complete sequences were reported from two patients, one from Philippines and the second from Brisbane (who had visited Malaysia, Thailand and Indonesia), which were different in 12 single nucleotide substitutions that resulted in changing 9 amino acids (18). Another study that conducted onto Indian isolates at the nucleotide level showed 48 point mutations along the length of the gene including 42 non-synonymous and six synonymous amino acid changes (19).

In our study nucleotide analysis showed that there were 11 SNP (single nucleotide polymorphism) along the gene that resulted in changes of six amino acid substitutions in comparison with S3 isolate reported from Indian (GenBank ACCESSION NO.: EF025195.1), while 21 point mutations and 13 changes in amino acids happened in compare to *SKO814*, Korean isolate, (GenBank ACCESSION NO.: GU476488.1). Additionally, 24 point mutations and 18 amino acid changes were observed in the reported isolate from El Salvador (GenBank ACCESSION NO.:XM_001615397). The ali-gnment between the Iranian isolate and Indian, Korean and El Salvador samples showed the range of 96%-99% homology.

As it has been illustrated in Fig. 2, the most variations among amino acids occurred in domain I in compare with Sal-1 isolate (from El Salvador). These changes can be resulted in diversity within parasite populations. So it is suggested that domain I could be applied to determine the varieties for parasite populations.

Domain II and domain III from Iranian isolate were completely similar to S3, but with one difference to SKO814 and Sal-1isolates in both domains (Fig. 2). These domains had the most identity in our study with the reported genes. Domain I and domain II can induce protective immunity and provide useful markers to detect the presence of antibodies in individuals who live in endemic areas (20, 21). So this region could be a good candidate for immunological researches and producing a protective vaccine. Domain III is responsible for cross-species reactivity of the antibody response to AMA-1 (5).

Based on the previous reports, the structure of PvAMA-1 is stabilized by 16 cysteine residues; these residues were totally conserved in the Iranian isolate (Fig. 2). Cysteines are necessary for constant conformation of related protein and antibody productions against this protein (22). In spite of the high similarity in domain II with the compared outside isolates, it is obvious that domain I in our study was different with other reported AMA-1 genes.

In conclusion, at the present study, we cloned and characterized three domains of PvAMA-1 gene from an Iranian patient. Predicted protein sequence of this gene obviously has a high degree of identity (99%) with strong homology to the PvAMA-1 gene of *P. vivax* with Asian isolates and also has 96% similarity with PvAMA-1 gene from El Salvador isolate****.
